# Tetra­aqua­bis(4-formyl­benzoato-κ*O*)cobalt(II) tetra­hydrate

**DOI:** 10.1107/S1600536808003140

**Published:** 2008-02-06

**Authors:** Zhao-Peng Deng, Shan Gao, Li-Hua Huo, Seik Weng Ng

**Affiliations:** a School of Chemistry and Materials Science, Heilongjiang University, Harbin 150080, People’s Republic of China; b Department of Chemistry, University of Malaya, 50603 Kuala Lumpur, Malaysia

## Abstract

The Co^II^ atom in the title compound, [Co(C_8_H_5_O_3_)_2_(H_2_O)_4_]·4H_2_O, which exists in an all-*trans* octa­hedral coordination geometry, lies on a center of inversion. The coordinated and uncoordinated water mol­ecules engage in extensive hydrogen-bonding inter­actions, forming a three-dimensional hydrogen-bonded network.

## Related literature

Hexaaqua­cobalt(II) bis­(4-formyl­benzoate) dihydrate was isolated from the reaction of cobalt(II) acetate and 4-formyl­benzoic acid in the presence of sodium hydroxide; see Deng *et al.* (2006*b* ▶). The reaction with pyridine in place of sodium hydroxide yielded the formylbenzoate-coordinated title compound. This is isostructural with the nickel analog; see Deng *et al.* (2006*a* ▶).
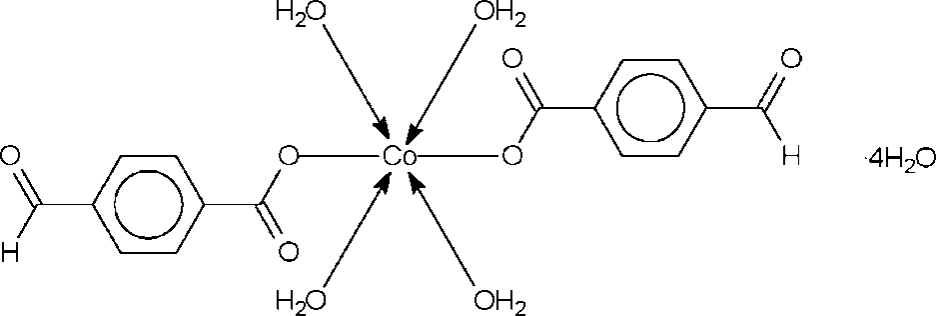


## Experimental

### 

#### Crystal data

[Co(C_8_H_5_O_3_)_2_(H_2_O)_4_]·4H_2_O
*M* *_r_* = 501.30
Triclinic, 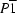

*a* = 7.1472 (3) Å
*b* = 7.4759 (4) Å
*c* = 11.5720 (6) Å
α = 77.114 (2)°
β = 77.905 (2)°
γ = 63.839 (1)°
*V* = 536.61 (5) Å^3^
*Z* = 1
Mo *K*α radiation
μ = 0.87 mm^−1^
*T* = 295 (2) K
0.30 × 0.26 × 0.22 mm

#### Data collection

Rigaku R-AXIS RAPID diffractometer
Absorption correction: multi-scan (*ABSCOR*; Higashi, 1995 ▶) *T*_min_ = 0.666, *T*_max_ = 0.832
5294 measured reflections
2426 independent reflections
2270 reflections with *I* > 2σ(*I*)
*R*_int_ = 0.015

#### Refinement

*R*[*F* ^2^ > 2σ(*F* ^2^)] = 0.027
*wR*(*F* ^2^) = 0.080
*S* = 1.03
2426 reflections
174 parameters
12 restraints
H atoms treated by a mixture of independent and constrained refinement
Δρ_max_ = 0.35 e Å^−3^
Δρ_min_ = −0.27 e Å^−3^

### 

Data collection: *RAPID-AUTO* (Rigaku, 1998 ▶); cell refinement: *RAPID-AUTO*; data reduction: *CrystalStructure* (Rigaku/MSC, 2002 ▶); method used to solve structure: atomic coordinates taken from the isostructural nickel analog; program(s) used to refine structure: *SHELXL97* (Sheldrick, 2008 ▶); molecular graphics: *X-SEED* (Barbour, 2001 ▶); software used to prepare material for publication: *publCIF* (Westrip, 2008 ▶).

## Supplementary Material

Crystal structure: contains datablocks global, I. DOI: 10.1107/S1600536808003140/bt2668sup1.cif

Structure factors: contains datablocks I. DOI: 10.1107/S1600536808003140/bt2668Isup2.hkl

Additional supplementary materials:  crystallographic information; 3D view; checkCIF report

## Figures and Tables

**Table 1:** Selected bond lengths (Å)

Co1—O1	2.098 (1)
Co1—O1*W*	2.113 (1)
Co1—O2*W*	2.116 (1)

**Table 2:** Hydrogen-bond geometry (Å, °)

*D*—H⋯*A*	*D*—H	H⋯*A*	*D*⋯*A*	*D*—H⋯*A*
O1*W*—H1*W*1⋯O3^i^	0.86 (1)	1.77 (1)	2.611 (2)	166 (2)
O1*W*—H1*W*2⋯O3*W*^ii^	0.84 (1)	2.11 (1)	2.925 (2)	161 (2)
O2*W*—H2*W*1⋯O3*W*	0.85 (1)	1.97 (1)	2.808 (2)	168 (2)
O2*W*—H2*W*2⋯O4*W*^ii^	0.85 (1)	1.97 (1)	2.808 (2)	169 (2)
O3*W*—H3*W*1⋯O4*W*	0.85 (1)	2.00 (1)	2.810 (2)	159 (2)
O3*W*—H3*W*2⋯O1^iii^	0.84 (1)	2.19 (1)	2.992 (2)	159 (2)
O4*W*—H4*W*1⋯O2^iv^	0.85 (1)	1.93 (1)	2.771 (2)	169 (3)
O4*W*—H4*W*2⋯O3^i^	0.85 (1)	2.00 (1)	2.841 (2)	172 (3)

Symmetry codes: (i) 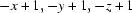; (ii) 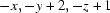; (iii) 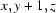; (iv) 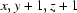.

**Fig. 1.**
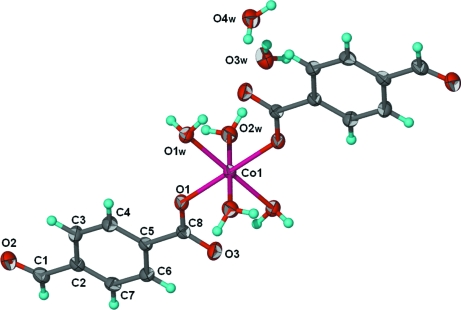
Anisotropic displacement parameter plot of (I). Displacement ellipsoids are drawn at the 50% probability level and H atoms as spheres of arbitrary radius.

## supplementary crystallographic information

### Comment

Hexaaquacobalt(II) bis(4-formylbenzoate) dihydrate was isolated from the reaction of cobalt(II) acetate and 4-formylbenzoic acid in the presence of sodium hydroxide (Deng *et al.*, 2006*b*). The reaction with pyridine in place of sodium hydroxide yielded the formybenzoate-coordinated title compound.

### Experimental

Cobalt diacetate dihydrate (2.32 g, 10 mmol) was added to an aqueous solution of 4-formylbenzoic acid (3.0 g, 20 mmol) that was earlier been treated with 1 ml pyridine to give a pH of 6. The solution was allowed to evaporate at room temperature; pink prismatic crystals separated from the filtered solution after several days. C&H elemental analysis. Calc. for C_16_H_26_O_14_Co: C 38.33, H 5.23%. Found: C 38.36, H 5.24%.

### Refinement

The carbon-bound H atoms were placed in calculated positions [C–H 0.93 Å and *U*_iso_(H) 1.2*U*_eq_(C)], and were included in the refinement in the riding-model approximation. The water H-atoms were located in a difference Fourier map, and were refined with distance restraints of O–H 0.85±0.01 Å and H···H 1.39±0.01 Å; their displacement parameters were freely refined.

### Crystal data

**Table:**

[Co(C_8_H_5_O_3_)_2_(H_2_O)_4_]·4H_2_O	*Z* = 1
*M**_r_* = 501.30	*F*_000_ = 261
Triclinic, *P*1	*D*_x_ = 1.551 Mg m^−^^3^
Hall symbol: -P 1	Mo *K*α radiation λ = 0.71073 Å
*a* = 7.1472 (3) Å	Cell parameters from 4983 reflections
*b* = 7.4759 (4) Å	θ = 3.1–27.5º
*c* = 11.5720 (6) Å	µ = 0.87 mm^−^^1^
α = 77.114 (2)º	*T* = 295 (2) K
β = 77.905 (2)º	Prism, pink
γ = 63.839 (1)º	0.30 × 0.26 × 0.22 mm
*V* = 536.61 (5) Å^3^	

### Data collection

**Table:**

Rigaku R-AXIS RAPID diffractometer	2426 independent reflections
Radiation source: fine-focus sealed tube	2270 reflections with *I* > 2σ(*I*)
Monochromator: graphite	*R*_int_ = 0.016
Detector resolution: 10.000 pixels mm^-1^	θ_max_ = 27.5º
*T* = 295(2) K	θ_min_ = 3.1º
ω scans	*h* = −9→9
Absorption correction: multi-scan(ABSCOR; Higashi, 1995)	*k* = −9→9
*T*_min_ = 0.666, *T*_max_ = 0.832	*l* = −15→14
5294 measured reflections	

### Refinement

**Table:**

Refinement on *F*^2^	Secondary atom site location: difference Fourier map
Least-squares matrix: full	Hydrogen site location: inferred from neighbouring sites
*R*[*F*^2^ > 2σ(*F*^2^)] = 0.027	H atoms treated by a mixture of independent and constrained refinement
*wR*(*F*^2^) = 0.080	*w* = 1/[σ^2^(*F*_o_^2^) + (0.0481*P*)^2^ + 0.1724*P*] where *P* = (*F*_o_^2^ + 2*F*_c_^2^)/3
*S* = 1.03	(Δ/σ)_max_ = 0.001
2426 reflections	Δρ_max_ = 0.36 e Å^−^^3^
174 parameters	Δρ_min_ = −0.27 e Å^−^^3^
12 restraints	Extinction correction: none
Primary atom site location: structure-invariant direct methods	

### Fractional atomic coordinates and isotropic or equivalent isotropic displacement parameters (Å^2^)

**Table:**

	*x*	*y*	*z*	*U*_iso_*/*U*_eq_	
Co1	0.5000	0.5000	0.5000	0.02806 (11)	
O1	0.45499 (18)	0.39037 (19)	0.36093 (10)	0.0359 (3)	
O2	0.0927 (2)	0.2392 (2)	−0.11561 (12)	0.0492 (3)	
O3	0.76754 (19)	0.3176 (2)	0.25021 (12)	0.0460 (3)	
O1W	0.19780 (18)	0.52308 (19)	0.58061 (11)	0.0381 (3)	
H1W1	0.192 (3)	0.570 (3)	0.6429 (14)	0.057 (7)*	
H1W2	0.091 (3)	0.602 (3)	0.5461 (19)	0.071 (8)*	
O2W	0.3680 (2)	0.79054 (18)	0.40003 (12)	0.0408 (3)	
H2W1	0.329 (4)	0.893 (3)	0.434 (2)	0.067 (8)*	
H2W2	0.271 (3)	0.807 (4)	0.362 (2)	0.069 (8)*	
O3W	0.2016 (3)	1.1588 (2)	0.48800 (14)	0.0544 (4)	
H3W1	0.152 (3)	1.158 (3)	0.5610 (10)	0.051 (6)*	
H3W2	0.282 (4)	1.218 (4)	0.470 (2)	0.096 (11)*	
O4W	−0.0173 (2)	1.1045 (2)	0.71542 (13)	0.0534 (4)	
H4W1	0.001 (4)	1.159 (3)	0.767 (2)	0.076 (8)*	
H4W2	0.048 (5)	0.9775 (14)	0.731 (3)	0.096 (11)*	
C1	0.2664 (3)	0.2311 (2)	−0.11986 (15)	0.0384 (4)	
H1	0.3563	0.2070	−0.1910	0.046*	
C2	0.3455 (2)	0.2570 (2)	−0.01950 (13)	0.0311 (3)	
C3	0.2157 (2)	0.3005 (3)	0.08787 (15)	0.0351 (3)	
H3	0.0779	0.3139	0.0967	0.042*	
C4	0.2921 (2)	0.3237 (3)	0.18106 (14)	0.0344 (3)	
H4	0.2052	0.3537	0.2525	0.041*	
C5	0.4989 (2)	0.3026 (2)	0.16869 (13)	0.0279 (3)	
C6	0.6284 (2)	0.2574 (2)	0.06149 (14)	0.0321 (3)	
H6	0.7668	0.2420	0.0528	0.039*	
C7	0.5514 (3)	0.2353 (2)	−0.03205 (14)	0.0334 (3)	
H7	0.6380	0.2059	−0.1037	0.040*	
C8	0.5803 (2)	0.3383 (2)	0.26758 (13)	0.0297 (3)	

### Atomic displacement parameters (Å^2^)

**Table:**

	*U*^11^	*U*^22^	*U*^33^	*U*^12^	*U*^13^	*U*^23^
Co1	0.02531 (16)	0.03279 (16)	0.02910 (16)	−0.01180 (12)	−0.00411 (10)	−0.01027 (11)
O1	0.0324 (5)	0.0492 (6)	0.0324 (6)	−0.0187 (5)	−0.0014 (4)	−0.0167 (5)
O2	0.0491 (8)	0.0561 (8)	0.0500 (8)	−0.0196 (6)	−0.0160 (6)	−0.0179 (6)
O3	0.0315 (6)	0.0714 (9)	0.0433 (7)	−0.0231 (6)	−0.0004 (5)	−0.0248 (6)
O1W	0.0298 (6)	0.0469 (7)	0.0409 (7)	−0.0161 (5)	−0.0034 (5)	−0.0134 (5)
O2W	0.0431 (7)	0.0362 (6)	0.0432 (7)	−0.0124 (5)	−0.0143 (5)	−0.0063 (5)
O3W	0.0666 (9)	0.0547 (8)	0.0556 (9)	−0.0350 (8)	−0.0125 (7)	−0.0084 (7)
O4W	0.0528 (8)	0.0562 (9)	0.0547 (9)	−0.0140 (7)	−0.0207 (6)	−0.0204 (7)
C1	0.0468 (9)	0.0372 (8)	0.0326 (8)	−0.0149 (7)	−0.0078 (7)	−0.0104 (6)
C2	0.0378 (8)	0.0278 (7)	0.0290 (7)	−0.0132 (6)	−0.0063 (6)	−0.0055 (5)
C3	0.0300 (7)	0.0459 (9)	0.0341 (8)	−0.0184 (7)	−0.0029 (6)	−0.0102 (6)
C4	0.0325 (8)	0.0448 (8)	0.0276 (7)	−0.0171 (7)	0.0008 (6)	−0.0106 (6)
C5	0.0300 (7)	0.0268 (6)	0.0278 (7)	−0.0114 (6)	−0.0048 (5)	−0.0056 (5)
C6	0.0292 (7)	0.0348 (7)	0.0331 (8)	−0.0137 (6)	−0.0007 (6)	−0.0084 (6)
C7	0.0379 (8)	0.0340 (7)	0.0273 (7)	−0.0143 (6)	0.0017 (6)	−0.0093 (6)
C8	0.0295 (7)	0.0297 (7)	0.0305 (7)	−0.0110 (6)	−0.0050 (6)	−0.0071 (5)

### Geometric parameters (Å, °)

**Table:**

Co1—O1	2.098 (1)	O4W—H4W1	0.847 (10)
Co1—O1^i^	2.098 (1)	O4W—H4W2	0.849 (10)
Co1—O1W^i^	2.113 (1)	C1—C2	1.475 (2)
Co1—O1w	2.113 (1)	C1—H1	0.9300
Co1—O2w	2.116 (1)	C2—C7	1.388 (2)
Co1—O2W^i^	2.116 (1)	C2—C3	1.393 (2)
O1—C8	1.2630 (18)	C3—C4	1.381 (2)
O2—C1	1.207 (2)	C3—H3	0.9300
O3—C8	1.2543 (19)	C4—C5	1.395 (2)
O1W—H1W1	0.858 (9)	C4—H4	0.9300
O1W—H1W2	0.844 (9)	C5—C6	1.393 (2)
O2W—H2W1	0.849 (9)	C5—C8	1.507 (2)
O2W—H2W2	0.848 (9)	C6—C7	1.382 (2)
O3W—H3W1	0.845 (9)	C6—H6	0.9300
O3W—H3W2	0.838 (10)	C7—H7	0.9300
			
O1—Co1—O1^i^	180.0	O2—C1—C2	124.23 (16)
O1—Co1—O1W^i^	93.12 (5)	O2—C1—H1	117.9
O1^i^—Co1—O1W^i^	86.88 (5)	C2—C1—H1	117.9
O1—Co1—O1W	86.88 (5)	C7—C2—C3	119.95 (14)
O1^i^—Co1—O1W	93.12 (5)	C7—C2—C1	119.56 (14)
O1W^i^—Co1—O1W	180.0	C3—C2—C1	120.49 (14)
O1—Co1—O2W	86.82 (5)	C4—C3—C2	119.79 (14)
O1^i^—Co1—O2W	93.18 (5)	C4—C3—H3	120.1
O1W^i^—Co1—O2W	89.23 (5)	C2—C3—H3	120.1
O1W—Co1—O2W	90.77 (5)	C3—C4—C5	120.42 (14)
O1—Co1—O2W^i^	93.18 (5)	C3—C4—H4	119.8
O1^i^—Co1—O2W^i^	86.82 (5)	C5—C4—H4	119.8
O1W^i^—Co1—O2W^i^	90.77 (5)	C6—C5—C4	119.52 (14)
O1W—Co1—O2W^i^	89.23 (5)	C6—C5—C8	119.64 (13)
O2W—Co1—O2W^i^	180.0	C4—C5—C8	120.77 (13)
C8—O1—Co1	127.37 (10)	C7—C6—C5	120.04 (14)
Co1—O1W—H1W1	97.7 (16)	C7—C6—H6	120.0
Co1—O1W—H1W2	120.0 (19)	C5—C6—H6	120.0
H1W1—O1W—H1W2	108.3 (14)	C6—C7—C2	120.27 (14)
Co1—O2W—H2W1	118.7 (17)	C6—C7—H7	119.9
Co1—O2W—H2W2	113.8 (17)	C2—C7—H7	119.9
H2W1—O2W—H2W2	108.9 (15)	O3—C8—O1	124.60 (14)
H3W1—O3W—H3W2	110.9 (15)	O3—C8—C5	117.39 (13)
H4W1—O4W—H4W2	109.2 (15)	O1—C8—C5	118.00 (13)
			
O1^i^—Co1—O1—C8	0(100)	C4—C5—C6—C7	0.5 (2)
O1W^i^—Co1—O1—C8	−0.75 (13)	C8—C5—C6—C7	−176.45 (13)
O1W—Co1—O1—C8	179.25 (13)	C5—C6—C7—C2	−0.4 (2)
O2W—Co1—O1—C8	88.31 (13)	C3—C2—C7—C6	−0.2 (2)
O2W^i^—Co1—O1—C8	−91.69 (13)	C1—C2—C7—C6	−179.45 (14)
O2—C1—C2—C7	176.99 (16)	Co1—O1—C8—O3	10.4 (2)
O2—C1—C2—C3	−2.3 (3)	Co1—O1—C8—C5	−168.27 (9)
C7—C2—C3—C4	0.6 (2)	C6—C5—C8—O3	−2.7 (2)
C1—C2—C3—C4	179.83 (15)	C4—C5—C8—O3	−179.67 (15)
C2—C3—C4—C5	−0.4 (2)	C6—C5—C8—O1	176.00 (14)
C3—C4—C5—C6	−0.1 (2)	C4—C5—C8—O1	−0.9 (2)
C3—C4—C5—C8	176.80 (15)		

Symmetry codes: (i) −*x*+1, −*y*+1, −*z*+1.

### Hydrogen-bond geometry (Å, °)

**Table:**

*D*—H···*A*	*D*—H	H···*A*	*D*···*A*	*D*—H···*A*
O1W—H1W1···O3^i^	0.86 (1)	1.77 (1)	2.611 (2)	166 (2)
O1W—H1W2···O3W^ii^	0.84 (1)	2.11 (1)	2.925 (2)	161 (2)
O2W—H2W1···O3W	0.85 (1)	1.97 (1)	2.808 (2)	168 (2)
O2W—H2W2···O4W^ii^	0.85 (1)	1.97 (1)	2.808 (2)	169 (2)
O3W—H3W1···O4W	0.85 (1)	2.00 (1)	2.810 (2)	159 (2)
O3W—H3W2···O1^iii^	0.84 (1)	2.19 (1)	2.992 (2)	159 (2)
O4W—H4W1···O2^iv^	0.85 (1)	1.93 (1)	2.771 (2)	169 (3)
O4W—H4W2···O3^i^	0.85 (1)	2.00 (1)	2.841 (2)	172 (3)

Symmetry codes: (i) −*x*+1, −*y*+1, −*z*+1; (ii) −*x*, −*y*+2, −*z*+1; (iii) *x*, *y*+1, *z*; (iv) *x*, *y*+1, *z*+1.
